# Artificial intelligence applications in long-term care facilities: a scoping review across planning, design, operation, and assessment

**DOI:** 10.3389/fpubh.2026.1974526

**Published:** 2026-10-19

**Authors:** Yu Chen, Xinlan Song, Ning Sun, Jianan Li, Zhi Qiu

**Affiliations:** 1 Institute of Architectural Design and Theoretical Research, College of Civil Engineering and Architecture, Zhejiang University, Hangzhou, China; 2 College of Architecture and Urban Planning, Beijing University of Technology, Beijing, China

**Keywords:** artificial intelligence, building lifecycle, generative design, intelligent operation, long-term care facilities, scoping review

## Abstract

**Introduction:**

Improving the quality of long-term care facilities (LTCFs) is critical to safeguarding the health, safety, dignity, and quality of life of older adults amid global population aging. However, traditional design methods for LTCFs rely heavily on static codes and empirical judgment, limiting their ability to respond to dynamic care needs and balance multiple objectives across lifecycle stages. To address these limitations, artificial intelligence (AI) offers a promising solution through dynamic optimization and intelligent decision-making.

**Methods:**

A scoping review was conducted to systematically map the applications of AI in LTCFs across four lifecycle stages: planning and site selection, architectural design, operation and management, and assessment and retrofit. Searches were performed in Web of Science, Scopus, Google Scholar, SAGE Journals, and CNKI for literature published between January 2010 and December 2025, resulting in the inclusion of 38 eligible publications. The review was guided by the methodological framework proposed by Arksey and O’Malley and reported in accordance with PRISMA-ScR.

**Results:**

Four main application pathways were identified, including prediction and evaluation, generation and evolution, sensing and recognition, and simulation and modeling. Specifically, AI was applied to site-selection evaluation and demand forecasting during planning; spatial layout generation and intelligent equipment integration during design; safety monitoring, behavior recognition, and environmental control during operation; and comfort and safety assessment and retrofit optimization during assessment. Research was concentrated in operation and management, whereas evidence on assessment and retrofit remained limited.

**Discussion:**

Overall, AI shows potential to shift LTCFs from static, fragmented, and experience-based practices toward more data-informed, adaptive, and person-centered care environments. However, implementation remains constrained by data quality and standardization, facility heterogeneity, system integration, equitable access, privacy, and ethical concerns. This review provides planners, architects, engineers, care providers, facility managers, and public health decision-makers with a technology roadmap and identifies priorities for using AI-enabled built environments to support high-quality older adult care and sustainable long-term care systems.

## 1 Introduction

Global population aging is one of the most significant demographic trends of the twenty-first century. By 2050, the proportion of the global population aged 65 and above is projected to increase to 16.5% (1), with the population aged 80 and above growing particularly rapidly. This poses serious challenges to the safety, health support, and care accessibility of the built environment. To address these challenges, the World Health Organization (WHO) has advocated the development of age-friendly environments and emphasized human-centered long-term care services (2). In this context, Long-Term Care Facilities (LTCFs) emerge as a core building type that provides professional care and supportive residential environments, serving as important residential settings for this vulnerable population. The environmental quality of LTCFs directly affects residents’ safety, health, and overall quality of life (3, 4).

However, current practices for LTCFs face multiple challenges. Many facilities renovated from existing buildings are constrained by their original structures and service models, making it difficult to meet high standards for accessibility and health-supportive environments. Even in new construction projects, insufficient forecasting during the design stage regarding dynamic operational needs, such as changes in residents’ functional abilities and the optimization of care workflows, frequently results in limited spatial flexibility, inefficient functional organization and circulation, and high energy consumption in building systems (e.g., heating, ventilation, and air conditioning (HVAC) and lighting) (5, 6). These shortcomings may compromise the quality of care and service efficiency, which in turn adversely affects residents’ well-being. The root of these issues lies in conventional architectural design and operation methods that rely heavily on static codes and empirical judgment. Such approaches have difficulty effectively addressing the personalized and dynamic evolution of user needs and fail to achieve a refined balance among multiple objectives including safety, comfort, energy efficiency, and cost.

In recent years, the rapid development of advanced digital technologies such as artificial intelligence (AI), the Internet of Things (IoT), and big data has provided new approaches to addressing these challenges. These technologies have been explored across various levels of age-friendly environments. At the community level, smart sensing pathways and adaptive street lighting systems can automatically adjust illumination according to older adults’ movement trajectories and environmental lighting conditions, thereby improving nighttime safety and age-friendliness in public spaces (7). Meanwhile, in senior care buildings such as community-based day care centers and activity rooms for older adults, AI-based building control systems have been introduced to optimize energy use while monitoring and regulating indoor environmental conditions in real time to meet the physiological needs of older adults (8). At the residential level, Ambient Assisted Living (AAL) integrates sensor networks, context-aware systems, and machine learning (ML) algorithms to create responsive smart environments, providing technological support for health monitoring, safety assurance, and assistance with daily living (9). At the institutional level, sensing and monitoring systems are increasingly being deployed in LTCFs to support safety monitoring, personalized care, and behavior analysis. The operational data collected in these settings also provide empirical evidence for understanding spatial use patterns and supporting optimized design.

Collectively, these studies indicate that AI technologies are gradually being embedded in environmental perception, data analysis, intelligent decision-making, and system optimization, thereby driving older adult care and the built environment toward smarter and more dynamically adaptive directions, while creating new opportunities to improve care quality. Existing reviews have examined this topic from several perspectives. In older adult care, reviews have primarily adopted care-service and technological-intervention perspectives, with particular emphasis on health monitoring, daily activities assistance, and independent living (10–18). Some reviews focusing on LTCFs have examined how AI and related smart technologies improve care quality and operational efficiency through monitoring, digital health, information management, and the integration of healthcare services, while also considering stakeholder acceptance. However, reviews that examine AI-enabled spatial optimization of LTCF environments remain scarce (19). In smart healthcare facilities, which shares certain environmental similarities with LTCFs, reviews have addressed the role of AI in improving healthcare efficiency and quality (20–22) and have begun to explore, from an architectural design perspective, how AI can contribute to hospital functional space planning, patient and staff flow optimization, and design generation (23–25). These reviews provide an important foundation, but they have mainly focused on specific technological applications or design issues at individual stages, with limited attention to the systematic role of AI across the built environment as a whole. Established building lifecycle theory typically describes the lifecycle as a sequence of stages including initiation, project definition, design, construction, commissioning, operation, maintenance, refurbishment, replacement, deconstruction and so on (26). Despite this comprehensive framing, AI applications in LTCFs remain fragmented across these stages. This fragmentation prevents operational data from feeding back into design and retrofit decisions, limiting the formation of a lifecycle-based optimization loop. Consequently, the potential of AI to enhance the overall building performance and comprehensive quality of LTCFs remains limited.

Adapting established building lifecycle frameworks to the specific characteristics of LTCFs, this review integrates the processes most relevant and important for AI applications into four stages: planning and site selection, architectural design, operation and management, and assessment and retrofit. This research specifically addresses three interrelated questions: (1) What AI applications and technical pathways have been investigated across these four key stages? (2) How is the existing evidence distributed across these stages, and which areas remain underexplored? (3) What implementation challenges and future research opportunities arise from the integration of AI into LTCFs? To address these questions, this paper systematically examines the research developments, technical pathways, and current AI applications, identifies areas of research concentration and key knowledge gaps, and outlines priorities for future research and practice. By synthesizing this evidence, this review provides planners, architects, engineers, facility managers, and public health decision-makers with a lifecycle-oriented roadmap for integrating data-driven methods into LTCFs. It also identifies priorities for using AI-enabled built environments to support high-quality older adult care and sustainable long-term care systems, while providing a research foundation for future studies to examine the potential effects of these approaches on quality of care and residents’ well-being.

## 2 Methodology

This scoping review was guided by the methodological framework proposed by Arksey and O’Malley (27), which was considered appropriate for mapping heterogeneous sources of evidence across four key lifecycle stages of LTCFs, various types of AI technologies, and diverse application scenarios. The literature search, screening, and selection procedures were conducted systematically, and the review was reported in accordance with PRISMA-ScR (28).

### 2.1 Search strategy

The scope of this review spans architecture, AI technology, and gerontology. To capture relevant evidence across these fields, a systematic literature search was conducted using Web of Science, Scopus, Google Scholar, SAGE Journals, and the China National Knowledge Infrastructure (CNKI). The search strategy combined AI-related keywords (e.g., “artificial intelligence,” “machine learning”), LTCF-related keywords (e.g., “long-term care facilities,” “nursing home”), and lifecycle-specific keywords (e.g., “planning,” “design,” “operation,” “assessment”) (Table 1). Detailed search strategies for each database are presented in Supplementary Table 1. The publication period spanned January 1, 2010, to December 31, 2025, and the search was conducted from January to March 2026.

**Table 1:** Keyword matrix.

Keywords	Synonyms
Artificial Intelligence	“AI” OR “machine learning” OR “deep learning” OR “computer vision” OR “smart” OR “ambient assisted living” OR “AAL”
Long-term Care Facilities	“nursing home” OR “senior care facilities” OR “elderly care facilities” OR “community-embedded elderly care facilities”
Planning	“location planning” OR “spatial distribution” OR “accessibility” OR “spatial equity”
Design	“spatial configuration” OR “spatial layout” OR “room layout” OR “interior design” OR “generative design”
Operation	“management” OR “fall detection” OR “activity recognition” OR “behavior recognition” OR “safety monitoring” OR “environmental control” OR “indoor environment”
Assessment and Retrofit	“evaluation” OR “safety assessment” OR “performance assessment” OR “post-occupancy evaluation” OR “POE” OR “age-friendly retrofitting” OR “renovation” OR “renewal” OR “refurbishment”

### 2.2 Inclusion and exclusion criteria

Following the PRISMA procedure, after removing duplicates, we screened titles and abstracts to determine the scope of eligible studies. A study was included in this review only when it met several key criteria.

1. Directly related to AI technologies. In this review, AI technologies are broadly defined as computational methods that exhibit at least one of the following capabilities: learning from data; adaptive, heuristic, evolutionary or swarm-based search; autonomous or semi-autonomous decision-making; reasoning under uncertainty; or generative capability. AI-related intelligent computational techniques were included only when their specific applications demonstrated such capabilities. General deterministic optimization methods, conventional numerical simulations, and traditional algorithms that merely execute predefined procedures were excluded, unless they were integrated with AI components;
2. Focusing on LTCFs as the primary study object;
3. Exploring AI applications across one or more stages of the LTCF lifecycle.

Since the topic of this review is a relatively new field of research with a limited number of exact matches, studies were also included if they only partially addressed AI technology and LTCFs. In addition, considering that some home-based care and healthcare settings share similarities with care scenarios in LTCFs, selected studies on AI applications in home-based or medical care environments were also included.

Studies were excluded from this review if they met any of the following criteria:

1. Presenting purely technical AI papers with no direct relevance to LTCFs, such as research dedicated to pure human-machine interaction or software design for eldercare service platforms.
2. Focusing only on home-based care without addressing specific architectural spaces.
3. Concentrating mainly on clinical medical applications or nursing theories.
4. Constituting non-academic publications, such as news reports and industry white papers.

### 2.3 Study selection, snowballing, and data extraction

Two researchers independently assessed the relevance and eligibility of the studies according to predefined inclusion and exclusion criteria, through a screening process that included removing duplicates, screening titles and abstracts, and reviewing full texts. To enhance the comprehensiveness of the search and identify potentially relevant studies, backward snowballing was subsequently conducted. Studies retrieved through the database search that met the inclusion criteria were used as seed articles, and their reference lists were manually screened by the research team to identify potentially relevant studies concerning AI and the built environment of LTCFs. These candidate articles were then cross-checked against the original database search results and screened at the title, abstract, and full-text levels using the same inclusion and exclusion criteria as those applied in the primary search. Data were extracted using a predesigned extraction form that included article title, authors and publication year, country or region, core method, data type, and main findings (see the Supplementary Table 2). Subsequently, three experts reviewed the extracted data. Disagreements among the researchers in literature classification and data extraction were resolved through multiple rounds of group discussion.

## 3 Results

From the initial 3,290 identified records, 234 titles and abstracts were deemed relevant. After applying the exclusion criteria, 34 full-text articles were identified as eligible. Through backward snowballing, 29 additional candidate articles were identified, and four met the inclusion criteria, bringing the total number of studies included in this review to 38 (Figure 1). The following sections analyze the research progress, technical pathways, and application status of AI technologies in LTCFs across four stages.

**Figure 1:**
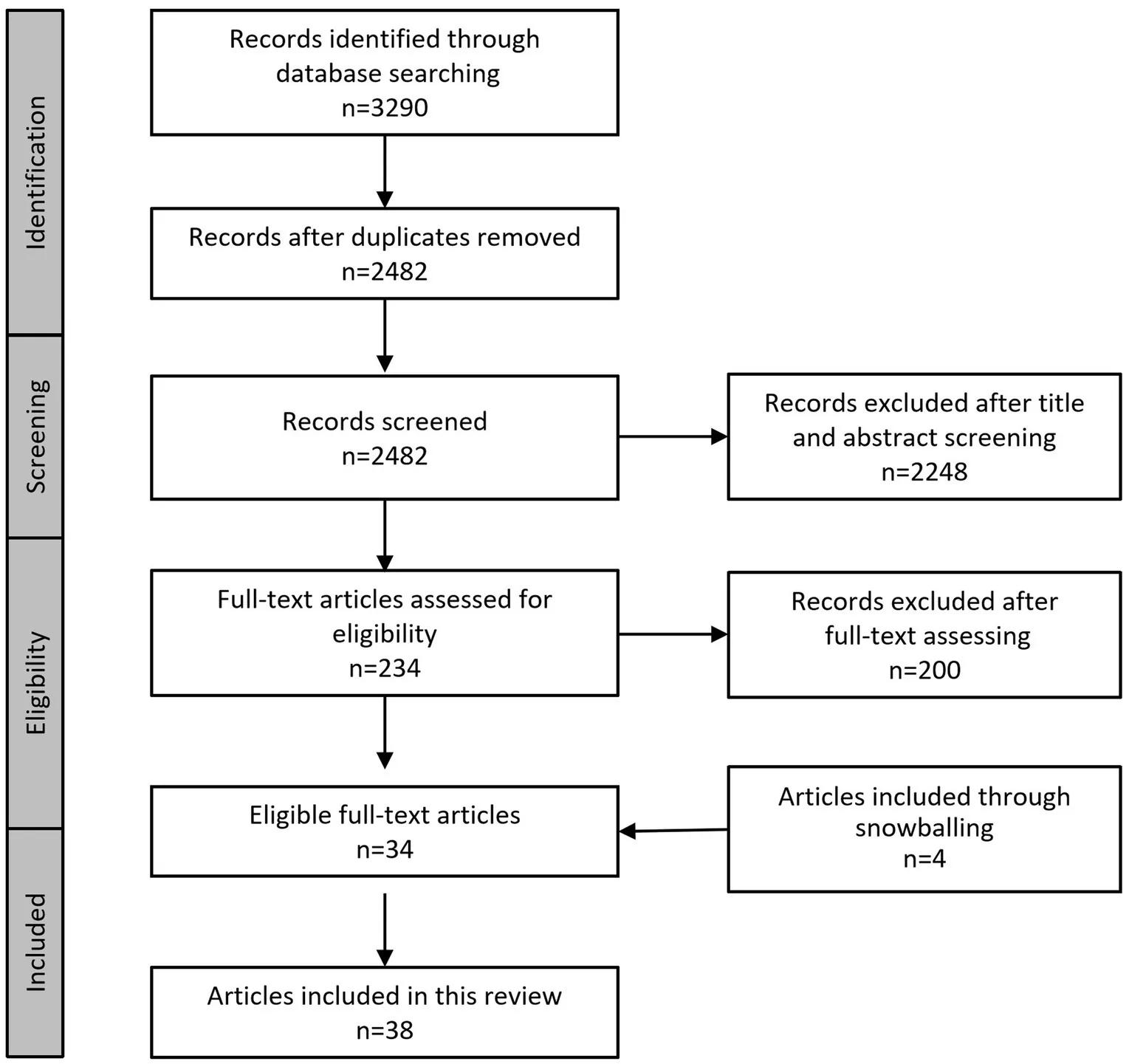
Study selection process (PRISMA-ScR flow diagram).

### 3.1 Planning and site selection

Site selection is a critical decision in the planning of LTCFs, as it directly affects service accessibility, spatial equity, resource allocation efficiency, and long-term operational performance (29). Conventional site selection studies are mostly based on static land-use planning and evaluation of existing conditions. Methods such as geographic information system (GIS) spatial analysis and the Two-Step Floating Catchment Area (2SFCA) method have often been used to assess spatial accessibility and distributional equity of facilities (30, 31). However, these methods struggle to capture dynamic changes in demographic structures or varying care needs. They also rarely integrate the complex influence of multiple stakeholders including governments, investors, operators, older adults, and families. With the introduction of AI technologies, site selection methods are shifting from static resource allocation toward intelligent decision-support systems that can simulate dynamic supply and demand and balance multiple objectives. Existing research can be summarized into four categories.

#### 3.1.1 Site suitability assessment based on multi-source data and ML

To address the limits of conventional approaches in quantifying multi-factor weights and handling nonlinear relationships, recent studies combine multi-source spatial data with ML to evaluate site suitability more flexibly. Point-of-interest (POI) data have been widely used because they can serve as proxies for the distribution of urban functions and service amenities (32, 33). ML models such as decision tree (DT) and random forest (RF) models demonstrate distinctive advantages in processing POI data and mining spatial association patterns (34, 35). For example, several studies have used the Iterative Dichotomiser 3 (ID3) decision tree algorithm to quantify the importance weights of POIs such as parks, hospitals, and supermarkets for LTCF site selection, and generated visual rule trees to intuitively identify suitable layout areas, thereby providing scientific evidence for site selection decisions (36–38). To further improve prediction accuracy and model stability, ensemble learning methods have been introduced. RF and Gradient Boosting Decision Tree (GBDT) models can process larger volumes of POI data and reduce overfitting risks through ensemble strategies (39). By dividing urban areas into fine-scale grid units, these models enable researchers to predict suitable locations and rank the relative importance of influencing factors. For instance, one study found that “distance to the nearest medical facility” and “older population density” were the two most critical factors affecting LTCF site selection (40). To improve the interpretability of such models, explainable artificial intelligence (XAI) methods such as Shapley Additive Explanations (SHAP) have been used to quantify how individual features such as population scale and policy factors contribute to predicted site-suitability outcomes. This helps reveal the complex mechanisms of multi-factor coupling and enhances the transparency and credibility of model decisions.

#### 3.1.2 Site selection decision-making under multi-objective coordination

Traditional multi-objective optimization methods for site selection such as weighted-sum and ε-constraint methods often rely on prior knowledge to determine weights, thereby transforming multi-objective problems into single-objective ones (41). These methods are highly subjective and usually provide only one optimal solution, making it difficult to analyze trade-offs among conflicting objectives. To overcome this limitation, researchers have proposed multi-objective optimization frameworks based on evolutionary algorithms (e.g., GAs and Particle Swarm Optimization, PSO) (42). These methods can simultaneously address multiple potentially conflicting objectives, including construction cost, service coverage, spatial accessibility, and environmental equity, and generate a Pareto-optimal front, which represents a set of solutions where no single objective can be further improved without degrading others. This allows decision-makers to visualize trade-offs among objectives and make more scientific and flexible decisions according to actual construction conditions and policy preferences, such as whether to prioritize maximum coverage or average accessibility under a fixed budget (43).

#### 3.1.3 Demand-oriented facility typology and differentiated allocation

The two categories of site selection approaches discussed above are primarily based on a supply-oriented perspective; however, according to public goods allocation theory, optimal supply should closely match actual demand (44). Accordingly, some studies adopt a demand-side perspective, using data analysis and ML to profile older populations and guide differentiated allocation. Regarding specific methodologies, these studies commonly use questionnaires, census data, or health databases to obtain information on older adults’ socioeconomic attributes, health status, and disability levels, and then apply clustering algorithms (e.g., K-means and spectral clustering) to identify groups of older adults with similar needs and map their spatial distributions. For example, one study integrated spectral clustering with unordered multi-class logistic regression to analyze the preferences of different groups of older adults. The findings quantified their specific choices regarding service content and facility types, such as comprehensive, medical-nursing integrated, and community-embedded facilities. It emphasized that facility allocation should be differentiated based on the payment capacity and medical care needs of different older adult populations. Ultimately, this approach establishes a demand-driven framework for facility classification and allocation (45).

#### 3.1.4 Supply–demand forecasting and long-term project planning involving multiple stakeholders

Aging is a dynamic process, and the supply–demand relationship of LTCFs is shaped by complex interactions among demographic migration, policy regulation, and market behavior. To address this dynamism and complexity, Agent-Based Modeling (ABM) has been introduced as a forward-looking planning tool. ABM can simulate the decision-making processes and outcomes of multiple stakeholders, including older adults (users), governments (policymakers), and operators (investors). It can project supply–demand dynamics of eldercare beds over future years (e.g., spatial mismatch and bed vacancy rates), thereby informing future resource allocation and policy intervention (46). For instance, one study simulated the layout of LTCFs in Shanghai for 2020–2030, assessed the existing layout, and suggested dynamic adjustment strategies, such as relieving pressure on central urban areas and optimizing bed structures over time (47). This method establishes site selection as a predictive planning process driven by future demand forecasting, thereby providing evidence-based support for the flexible planning of urban eldercare resources.

### 3.2 Architectural design

During the architectural design stage, traditional LTCF design relies heavily on general design codes, which can easily lead to fixed layout paradigms that are difficult to adapt to the personalized needs of older adults. Such limitations may result in inadequate environmental comfort, poorly coordinated functional adjacencies, and redundant care circulations. The introduction of AI offers a new approach for bypassing empirical and time-consuming traditional design workflows. It allows designers to rapidly generate and optimize diverse design alternatives while ensuring compliance and practicality, thereby supporting deeper spatial exploration and decision-making. Existing research can be summarized into three main directions.

#### 3.2.1 Automated design based on algorithmic generation and performance optimization

To improve design efficiency and support more evidence-based design decision-making, researchers have primarily explored the automated generation and optimization of architectural floor plans through two technical approaches. The first is optimization-driven generative design based on search algorithms such as GAs and PSO. By encoding design objectives and constraints as algorithmic rules and parameters, these methods can automatically search for and iteratively generate layouts that satisfy predefined conditions (48, 49). For example, one study used the “shortest care circulation” and “maximized natural daylighting” as dual objectives to generate one-story layouts using a GA and pathfinding algorithms (50). Another study used path planning algorithms to simulate the movement trajectories of older adults using assistive devices, precisely evaluating and optimizing accessibility and convenience among functional areas (51). Compared with conventional design methods, such approaches are more time- and cost-efficient. However, these algorithms often face difficulties in parameter tuning and may converge to local optima. The second pathway is deep-learning-based floor-plan generation, represented by Conditional Generative Adversarial Networks (CGANs). By learning topological patterns from existing high-quality cases, such models can generate preliminary zoning layouts or floor-plan proposals for long-term care spaces while accounting for design constraints and regulations (52, 53). Moreover, they can respond quickly to dynamic changes in needs and constraints (54). Nevertheless, both parametric optimization and generative AI still produce imprecise or unreasonable design elements. In this sense, they should be used as decision-support tools that expand the design search space and improve work efficiency, rather than as substitutes for architectural judgment. The evaluation, selection, and refinement of final design schemes still depend heavily on designers’ creative thinking and professional judgment.

#### 3.2.2 Spatial layout responding to psychological and social needs

Due to physical decline and reduced social connectedness, older adults are often more sensitive to the physical and social qualities of their living environments (55). Therefore, promoting positive social interaction and supporting psychological well-being are also central goals of LTCF design. AI technologies provide new methods for quantitatively evaluating and optimizing the social-supportive performance of spaces through multimodal sensing and behavior analysis. Specifically, sensor networks, radio-frequency identification (RFID) systems, and computer vision can be used to collect long-term and relatively unobtrusive data on residents’ movement trajectories, space-use frequency, and dwell time. These behavioral data can help identify the relationship between spatial configuration and social contact, and can inform the location, scale, and connectivity of communal spaces and social nodes. Meanwhile, abnormal behavior patterns (e.g., social isolation and wandering) may also serve as warning indicators of loneliness, cognitive decline, or unmet care needs. In addition, methods such as space syntax can be combined with AI-based analytical tools to quantify morphological indicators such as spatial integration and visibility, which makes it possible to link spatial form with social behavior and user experience, thereby providing evidence for the design of socially supportive environments (56, 57).

#### 3.2.3 Coordinated integration of intelligent devices and interior spaces

In many conventional LTCFs, smart devices are installed only after the building has been designed or occupied, which may lead to exposed equipment, cluttered wiring, and a poor fit between technology, spatial functions, and user experience. The introduction of AI and digital technologies is shifting intelligent device integration from passive adaptation toward spatial coordination. The key advancement lies in the ability to optimize equipment and space in an integrated manner through algorithms at the early design stage. Current applications are mainly reflected in two aspects. First, optimization algorithms can support the automated configuration of device layouts. For example, device functions (e.g., sensing range and signal strength), spatial constraints (e.g., visual occlusion and furniture layout), and privacy-sensitive requirements can be parameterized together to identify appropriate locations for sensors, call buttons, and other monitoring devices, thereby achieving reliable coverage with minimal intrusion (58). Second, simulation-based evaluation can be used to test the integrated performance of devices and space before implementation. By combining building information modeling (BIM) with agent-based simulation (ABS), it is possible to rehearse the interaction between device systems and older adults’ daily activities in a virtual environment and evaluate the potential impact of different integration strategies on care response efficiency, energy consumption, and even social atmosphere. This provides data support for design decision-making. For example, one study used a grey-box Bayesian optimization algorithm and introduced an indoor spatial simulator to combine a concrete facility space model with residents’ daily activity schedules. This approach simulated spatial-use patterns and sensor trigger trajectories in a virtual environment. These simulated data establish the foundation for a sample-efficient framework, optimizing motion sensor placement to support accurate activity recognition without compromising resident privacy in LTCFs (59). In this way, smart devices are no longer merely appended to space; rather, they become integrated components co-developed with spatial organization, supporting more accurate, unobtrusive, and user-oriented smart care environments.

### 3.3 Operation and management

LTCFs require sustained long-term operation, and operational quality directly affects both facility sustainability and residents’ quality of life. Traditional facilities often rely on manual management and experience-based decision-making, which can lead to delayed responses. AI technologies are enabling smarter operational models characterized by real-time sensing, dynamic optimization, and proactive intervention through continuous analysis of building environments and user behaviors. Existing applications during this stage can be summarized into two main directions.

#### 3.3.1 Spatial safety monitoring and risk prevention based on environmental sensing

Ensuring residents’ safety is the primary objective of LTCF operation, and fall prevention is particularly critical. Conventional monitoring methods, such as wearable devices and video surveillance, have limitations including inconvenience, privacy intrusion, and blind spots (60). AI-driven sensing systems provide alternative solutions through multimodal sensor fusion and non-intrusive monitoring (61). Typical applications include the use of embedded pressure sensor arrays (e.g., smart flooring) to continuously monitor gait, location, and posture information, enabling high-accuracy real-time fall detection and alarms through ML models without compromising privacy (62, 63). Other studies use depth images and thermal imaging data to continuously monitor older adults, applying computer vision to detect abnormal behaviors, such as prolonged dwelling or intense movement, and generating heat maps and activity frequency reports that reflect spatial use patterns for long-term health risk assessment (64).

#### 3.3.2 Data-driven intelligent control of the building environment

AI plays an important role in maintaining a healthy, comfortable, and energy-efficient indoor environment in LTCFs. By integrating various sensor modules, including those for temperature, humidity, lighting, air quality, and acoustics, smart living environment systems can monitor the indoor physical environment in real time and collect data, laying the foundation for precise control. The application primarily unfolds on two levels. The first level concerns predictive control of the physical environment. Conventional environmental control systems usually operate based on fixed setpoints or simple feedback mechanisms, making it difficult to adapt to dynamic outdoor weather conditions and interior occupancy changes. AI models, particularly ML methods such as Artificial Neural Networks (ANNs), can learn from historical data and model complex nonlinear relationships between building environmental responses and energy consumption, thereby enabling forward-looking optimization. For example, one study based on monitored data from nursing homes developed ANN models that use building features and indoor–outdoor environmental parameters as input variables to predict cooling demand. Such models can inform HVAC operation strategies that reduce energy consumption while maintaining thermal comfort for older residents (65). The second level concerns integrated control based on both physiological and psychological feedback. This approach goes beyond the control of single physical parameters and focuses on achieving “human-centered” adaptive environmental regulation through multimodal sensing (66). For example, an intelligent emotion analysis system based on a CNN and Laboratory Virtual Instrument Engineering Workbench (LabVIEW) has been developed for long-term care applications. By analyzing facial micro-expressions, the system can infer older adults’ emotional states in real time (67). When negative emotions such as anxiety or discomfort are identified, or when abnormal environmental parameters are detected, the system can automatically trigger linked adjustments, such as playing soothing music, regulating lighting color temperature and illuminance, or adjusting air-conditioning temperature. This demonstrates a technical pathway for linking real-time emotion recognition with environmental control.

### 3.4 Assessment and retrofit

Throughout the entire lifecycle of LTCFs, continuous assessment and targeted retrofit are essential for maintaining long-term suitability, environmental performance, and residents’ well-being (68, 69). Conventional post-occupancy evaluation (POE) mainly relies on periodic questionnaires and interviews, which are often delayed, subjective, and unable to capture dynamic behaviors. The introduction of AI has enhanced the continuity, objectivity, and real-time responsiveness of assessment processes. Existing studies during this stage mainly focus on the following three areas.

#### 3.4.1 Multi-sensory assessment of physical environmental comfort

Thermal, visual, acoustic, and air-quality-related comfort are important factors affecting indoor environmental quality (IEQ) (70), and their assessment is a core component of POE in LTCFs. Conventional methods struggle to continuously monitor the relationship between environmental parameters and older adults’ actual comfort perceptions. By integrating environmental sensing networks and computer vision technologies, AI enables synchronous monitoring of the physical environment and residents’ behaviors (71), while providing real-time comfort feedback (72). For example, one study employed ML algorithms to combine objective environmental data including air temperature, relative humidity, indoor air quality, illuminance, and noise level, with older adults’ subjective satisfaction votes. This model accurately quantified the contribution of each environmental factor to overall satisfaction, identified key influencing factors (e.g., temperature and noise) and potentially unsuitable spaces, and subsequently provided optimization recommendations (73). This data-driven dynamic evaluation model transforms POE into a continuous monitoring process, providing real-time and objective evidence to support precise operation and ongoing improvement of LTCFs. Moreover, such feedback data can be fed back as empirical parameters into the planning and design of new projects, forming an intelligent optimization loop across the building lifecycle.

#### 3.4.2 Spatial safety assessment for risk prevention and control

Spatial safety is another important focus of LTCF assessments. Conventional risk assessment is often based on expert judgment and qualitative inspection, making it difficult to systematically quantify and compare risks across different spatial conditions (74). By introducing algorithms such as fuzzy reasoning and neural networks, AI provides a more structured approach to evaluating complex spatial risk factors. For example, Gökler et al. proposed a hybrid risk assessment method that integrates the Fine-Kinney method with an ANFIS. In this framework, expert assessments of risk probability, frequency, and severity are transformed into training data, allowing the model to learn the relationship between spatial hazards and risk classes. Such an approach can be used to evaluate risks related to thresholds, insufficient corridor width, slippery surfaces, poor ergonomic conditions, and other hazardous spatial features in nursing homes (75). This model efficiently outputs the overall risk level of architectural spaces and the distribution of specific risk points. It successfully upgrades safety assessments from traditional qualitative inspections to data- and rule-based intelligent diagnostics, thereby significantly enhancing the rationality and precision of subsequent facility retrofits.

#### 3.4.3 Optimization of facility retrofit plans under multi-objective trade-offs

Retrofitting existing facilities often involves complex trade-offs among comfort improvement, energy reduction, and cost control, which conventional methods are unable to optimize systematically (76). AI optimization algorithms provide powerful decision-support tools for this purpose (77, 78). For example, Zhang et al. (79) used Non-Dominated Sorting Genetic Algorithm II (NSGA-II) to construct an evaluation framework that simultaneously simulates building energy consumption, indoor thermal comfort, and retrofit cost-effectiveness. The framework can automatically generate a large number of retrofit scenarios (e.g., envelope upgrades and system improvements) and calculate a Pareto-optimal solution set that characterizes the trade-offs among different objectives. Combined with cluster analysis, these solutions can be grouped into different strategy types, such as low-cost, comfort-oriented, or balanced retrofit options. This approach provides decision-makers with clear visual evidence, enabling them to scientifically select retrofit strategies that achieve the best balance among multiple objectives according to actual budget constraints and performance preferences, thereby realizing data-based multi-objective optimization.

### 3.5 Distribution and limitations of existing research fields

The preceding sections reviewed AI applications in the planning, design, operation, and assessment stages of LTCFs. The findings indicate that current research spans a diverse range of application scenarios and technical approaches. To better summarize which technologies are actually used, the AI technologies identified in the included literature were grouped into four application pathways according to their algorithmic logic and application objectives: prediction and evaluation, generation and evolution, sensing and recognition, and simulation and modeling. Figure 2 presents the main technology types used at each lifecycle stage.

**Figure 2:**
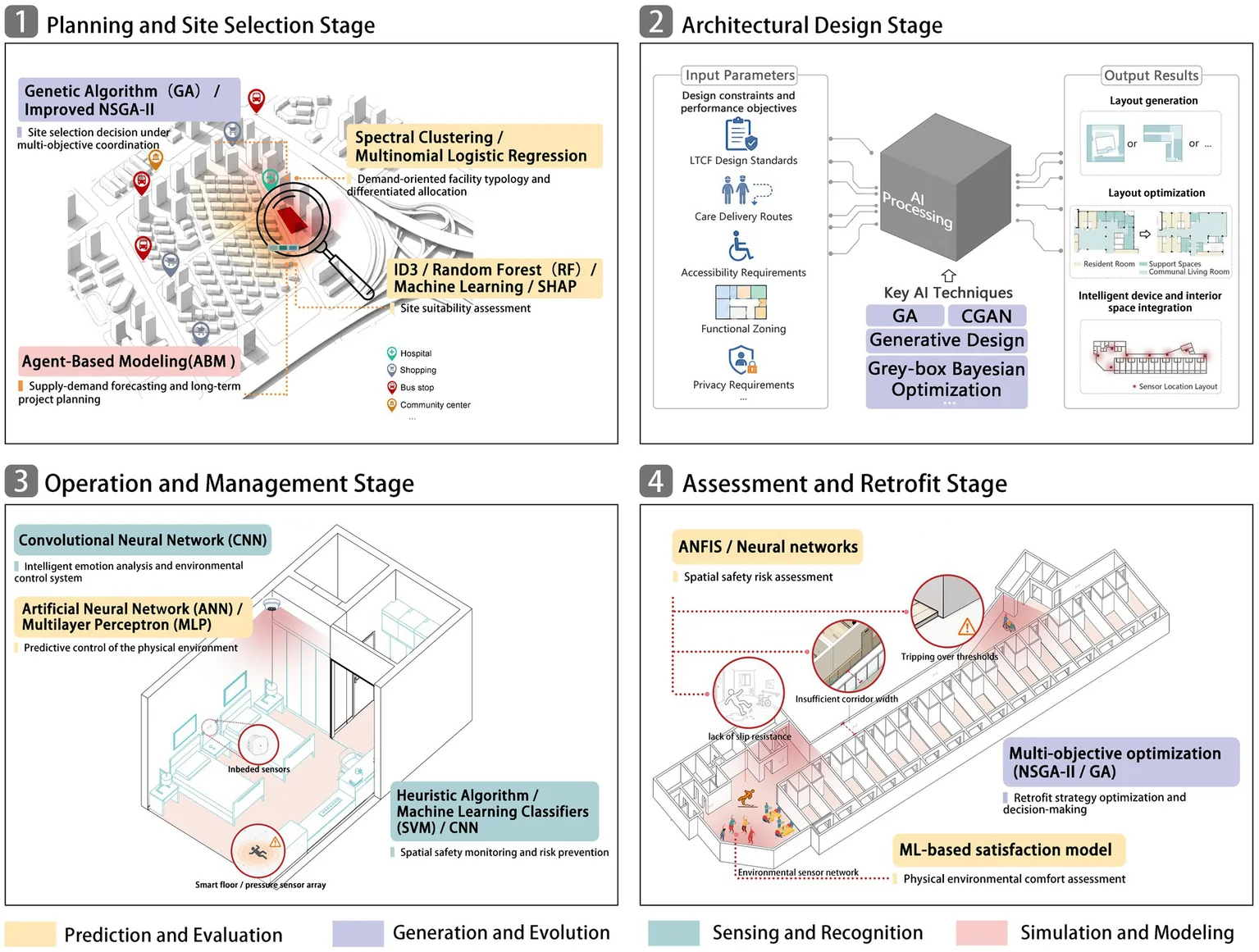
AI technologies applied across the lifecycle of LTCFs. The four panels correspond to the four lifecycle stages. The colored backgrounds of the technology labels indicate their corresponding AI application pathways, as defined in the legend: prediction and evaluation, generation and evolution, sensing and recognition, and simulation and modeling.

Specifically, the AI application areas identified in the included literature were thematically categorized, and a frequency analysis was conducted to compare research emphasis across lifecycle stages and application domains within each stage (Figure 3). The results indicate that the operation stage represents the most concentrated area of current research. In this stage, studies particularly focus on applications related to fall detection, non-intrusive sensor integration, abnormal behavior detection, post-construction safety monitoring, and routine risk prevention. Research in the planning and design stages mainly focuses on decision-making and scheme generation, such as site assessment, supply–demand forecasting, and spatial layout optimization. In contrast, research in the assessment stage remains relatively limited, mainly involving environmental comfort evaluation, safety assessment, and retrofit optimization.

**Figure 3:**
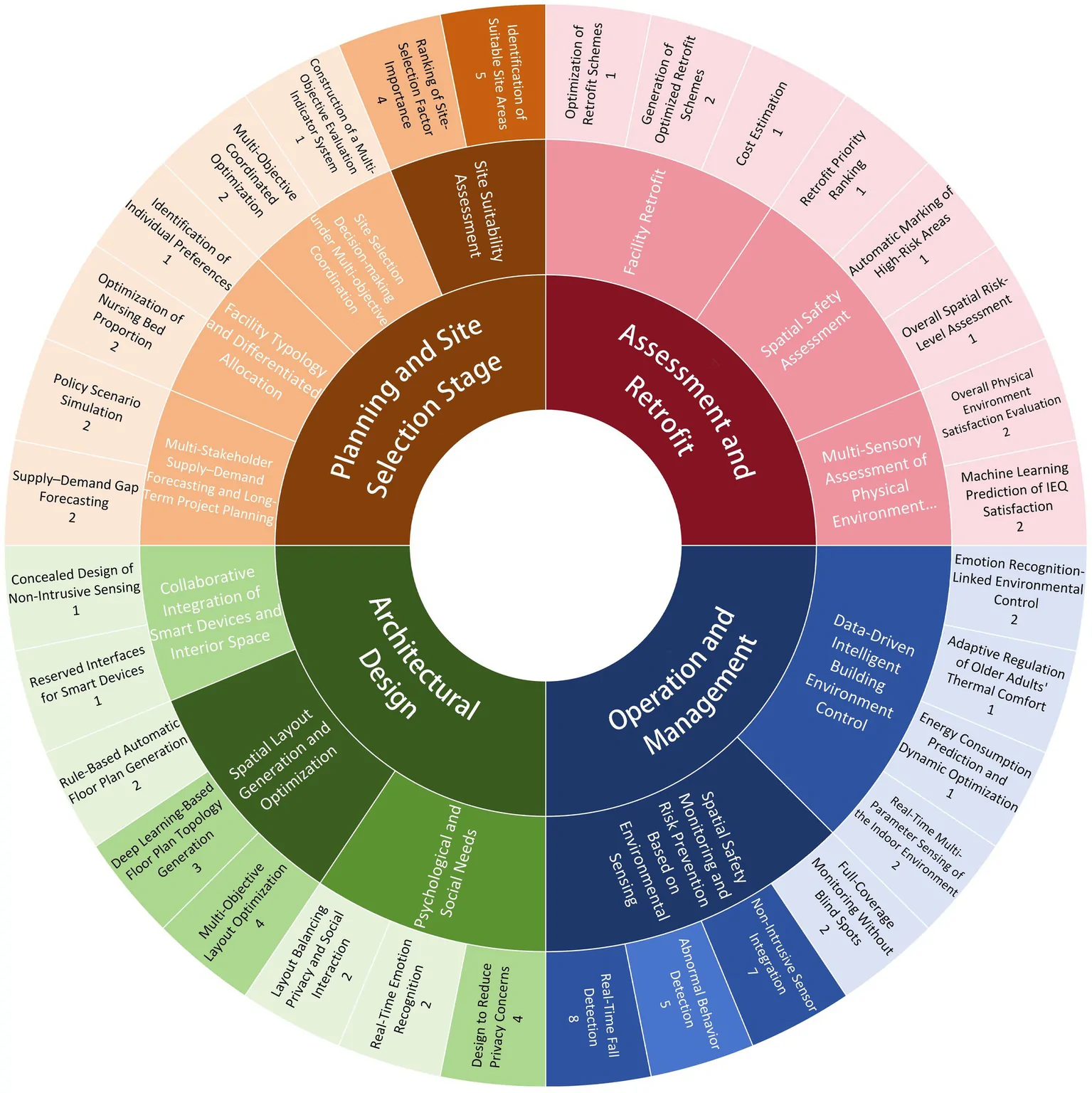
AI application hotspots across the LTCF lifecycle based on included studies. This figure employs a three-layer concentric structure, corresponding to the lifecycle stages, application domains, and specific application points. Different color schemes are used to distinguish the four lifecycle stages, while the shades within each color scheme indicate the frequency with which related topics are mentioned in the included literature; the darker the color, the greater the attention that topic has received in existing research. The numbers in the outermost ring are frequency data. In the statistical process, only studies focusing entirely on a specific application direction, or discussed in a dedicated section or paragraph, are counted in the category; if a single document addresses multiple application directions, it is counted separately for each.

## 4 Discussion

### 4.1 Impact of AI on architectural practice paradigms of LTCFs

The findings across the four lifecycle stages suggest that the coordinated application of these technologies may collectively empower the full lifecycle of LTCF development, reshape architectural practice paradigms, and foster a closer connection between the built environment and care practices.

First, AI is shifting LTCF design from reliance on established standards toward dynamic optimization. Conventional design methods rely heavily on standardized regulations and are often oriented toward universality. Although these methods help ensure basic safety and regulatory compliance, they face challenges in addressing the diverse needs of older adults as their physical functions change and their lifestyles evolve, while also failing to fully accommodate the work requirements of care staff. Generative and evolutionary AI methods such as GANs and multi-objective optimization algorithms help designers explore and optimize large-scale design alternatives regarding spatial layouts, care circulation and environmental parameters within regulatory frameworks. Meanwhile, sensing and recognition AI (e.g., computer vision and sensor networks) can continuously collect environmental and behavioral data during the operational stage. When combined with predictive and evaluative AI methods, such as ML models, these technologies can form a real-time feedback loop of “monitoring–assessment–optimization.” This transforms buildings from static products that are “finalized upon completion” into structures that can be continuously optimized based on real-use feedback (80).

Second, AI is transforming decision-making in LTCFs from experience-based, qualitative judgment toward data-driven, quantitative assessment. Major decisions such as site selection and retrofit have long relied on expert experience and qualitative judgment, which are subjective and difficult to reconcile when multiple objectives conflict. Predictive and evaluative AI, such as ID3 decision trees, RF and SHAP can use multisource data to identify relationships among complex variables and transform site suitability, spatial safety risks, and environmental satisfaction into quantifiable and comparable outputs, helping stakeholders better understand multifactor relationships and supporting more transparent decision-making. As noted above, sensing and recognition AI, combined with predictive models, can further provide empirical evidence on spatial performance to inform retrofit decisions.

Third, AI can help balance the needs of different user and stakeholder groups. Traditional facility planning has often centered on a limited set of objectives, such as cost, accessibility, or service efficiency, making it difficult to fully represent the conflicts and trade-offs among the interests of different stakeholders. For example, governments tend to emphasize the efficiency of public resource allocation, service coverage, and spatial equity; investors and operators must consider construction and operating costs, facility utilization, and long-term operational viability; while older adults and their families are generally more concerned with accessibility to transportation and healthcare services as well as their actual care needs. Evolutionary computation methods, particularly multi-objective evolutionary algorithms, simultaneously incorporate often-conflicting objectives (e.g., accessibility, equity, comfort, energy efficiency, and cost) into a computational framework, automatically generating visualizations of Pareto-optimal solution sets to provide a basis for decision-makers to compare and reconcile competing priorities. In addition, simulation- and modeling-based AI approaches (e.g., ABS) can construct digital models to quantitatively infer long-term supply–demand dynamics and benefits under different policy or market scenarios.

Fourth, AI enables a more integrated feedback mechanism across the lifecycle of LTCFs. In conventional processes, stages such as planning, design, operations, and evaluation are often separated, preventing valuable operational data from being effectively fed back into the system. The synergy of the four categories of AI technologies mentioned above helps break down this barrier. Real-time operational data collected by sensing-based AI can serve as input for evaluation models to diagnose performance problems and identify spatial or environmental risks. The resulting assessment outcomes and optimization objectives can then be translated into new rules, parameters, or constraints for generative and simulation-based AI, thereby informing future design or retrofit scenarios. Through this process, AI can connect different lifecycle stages and support an intelligent loop of “design–operation–assessment–redesign,” enabling LTCFs to adapt and improve throughout their service life.

### 4.2 Practical implications

The AI technologies discussed in this review are exerting a profound influence on professional practice and industrial development in the field of LTCFs.

At the level of architectural design, AI expands the professional boundaries of architects (81). Although it has long been emphasized that architects’ professional responsibilities should span the entire lifecycle of a building, in practice, due to a lack of mature standards and market practices, they are often limited to the design phase. The introduction of AI may help break this limitation. Specifically, architects can leverage predictive models to conduct data-driven demand analysis and project positioning, utilize generative design and performance simulation tools to draft and optimize schemes, and employ intelligent POE models to participate in performance diagnosis, spatial adjustment, and retrofit decision-making. Furthermore, AI translates the long-advocated “human-centered” architectural design philosophy into actionable practices (82). In LTCFs, this philosophy is challenging to fully implement in actual operations due to the individual variations in older adults’ needs and the dynamic shifts in care requirements. Today, AI technologies, such as multimodal sensing and affective computing, can continuously and unobtrusively collect data on residents’ physiological indicators, behavioral patterns, and emotional states, transforming the abstract concept of “experience” into analyzable data. This makes it possible to create proactive, “human-centered” environments (83, 84).

At the level of older adult care practice, by closely connecting the spatial environment, risk monitoring, and care activities, AI is reshaping the operational modes of long-term care services and supporting more personalized approaches to care. For example, addressing the pain points of traditional manual room rounds involving blind spots, delayed responses, and high labor costs, unobtrusive monitoring systems based on computer vision and millimeter-wave radar can provide 24/7, high-accuracy fall detection and early warnings of abnormal vital signs. Such systems can shift care from “passive response” toward “active prevention,” thereby reducing operational risks and improving response efficiency. Against the background of persistent labor shortages, AI and robotics may be able to undertake some repetitive and physically demanding care tasks, such as assisted bathing and rehabilitation training, thereby reducing staff pressure and improving service standardization (85). This allows limited human resources to be devoted more to activities that are difficult for technology to replace, such as communication, companionship, and advanced care.

At the public health system level, the value of AI also lies in supporting the coordinated allocation of long-term care resources and the synergy of service systems. Long-term care needs are characterized by substantial geographical disparities and dynamic changes. AI tools such as predictive analytics and demand simulation can assist decision-makers in systematically identifying service gaps across different areas and optimizing the spatial distribution of facilities and resource allocation strategies, thereby improving the spatial accessibility of services and supporting greater equity in resource distribution. Additionally, some LTCFs are beginning to explore digital twins and integrated operation platforms to connect dispersed smart devices, care tasks, and resource information, thereby achieving closed-loop management of care processes and dynamic, intelligent resource allocation. Such integrated systems not only have the potential to improve information integration and resource coordination within individual facilities, but may also lay the foundation for developing an integrated continuum of care that coordinates resources across institutional, community-based, and home-based care settings.

### 4.3 Limitations and challenges of existing AI technologies

Although AI technologies show great potential across the full lifecycle of LTCFs, current research and practice still face a series of unresolved challenges at the technical, data, ethical, and implementation levels when responding to complex real-world conditions (86, 87).

First, there are issues related to data quality and standardization. The performance of AI models depends heavily on high-quality training data, yet the current data infrastructure for LTCFs has significant shortcomings. Macro-level data used for planning analysis (e.g., POI and demographic data) may be incomplete or updated slowly in newly developed areas and rural regions, which directly affects the accuracy of site selection and demand prediction. During the design and assessment stages, the data used by AI models, including architectural drawings and environmental monitoring records, often lack clear classification of key attributes such as facility type and care level, making it difficult for machines to understand and use the data effectively. In addition, fine-grained data on older adults’ behaviors, preferences, emotions, and subjective experiences are difficult to collect at scale in a standardized way. Therefore, building high-quality, standardized, and lifecycle-oriented LTCF databases is a key foundation for future AI applications and warrants further exploration.

Second, there is a contradiction between the complexity and heterogeneity of LTCFs and the tendency of AI models to pursue standardized and generalizable patterns. LTCFs are not homogeneous products. They differ in operational models, care recipients, and service philosophies. However, many generative design and layout optimization studies tend to treat LTCFs as a single category and extract “generic modules” or “average patterns” from mixed and coarse-grained datasets. As a result, algorithms may fail to capture individual preferences, specific care workflows, and dynamic operational needs. This can lead to generated schemes that deviate from real practice and still require substantial manual correction, thereby limiting the practical effectiveness and credibility of AI-supported design. Future research should therefore focus on how to extract standardized data structures from complex real-world conditions while still preserving the specific needs and operational characteristics of different LTCFs.

Third, the large-scale implementation of AI faces challenges related to system integration and long-term adaptability. The problem of “data silos” is prominent. Older adults’ health data, behavior data, and building environmental data are often stored in different systems with inconsistent standards and poor interoperability, thereby hindering comprehensive AI applications based on the fusion of multi-source information (88, 89). More importantly, smart devices and AI software are updated over months or years, whereas buildings often have service lives of several decades. This mismatch may cause high-cost systems deployed in the early stages to become outdated quickly. Later upgrades can also be expensive and may result in exposed equipment, disordered cabling, visual disruption, or insufficient space for device installation and robot movement. Promoting the integrated co-design of AI systems and architectural spaces is therefore essential. At the architectural design stage, for example, space can be reserved for conduits, cabling, and equipment installation, while future operational needs, such as sensor replacement and equipment maintenance, should also be taken into account. In terms of digital and system integration, interoperability among different devices and platforms can be enhanced through the adoption of standardized data interfaces and semantic models wherever possible. BIM and digital twins can further serve as an information backbone spanning the design, operation, and retrofit stages, allowing updated smart devices to be progressively integrated into existing systems and thereby enhancing the technological adaptability of the building.

Fourth, there are issues of equity and universality in application. Current research and practice on AI applications show significant regional and resource imbalances, with most efforts concentrated in developed regions with well-established infrastructure and ample funding, or in newly built, high-end LTCF projects. A notable contradiction is that grassroots and retrofitted facilities, characterized by aging architecture, spatial constraints, and strained caregiving workforces, often have the greatest need for intelligent solutions to compensate for hardware deficiencies and improve safety and care efficiency. However, such facilities often face tight budgets and struggle to afford the deployment of today’s complex, mainstream systems. Therefore, developing and promoting low-cost, easy-to-deploy “lightweight” AI solutions, or exploring indirect sensing technologies based on existing infrastructure, such as Wi-Fi and conventional cameras, has become a critical task. By lowering technological barriers, such approaches can enable a broader population of older adults to benefit more equitably from technological advances (90, 91).

Fifth, data privacy and ethical challenges remain critical. Deploying sensing and monitoring AI in LTCFs inevitably involves trade-offs among privacy, dignity, safety, and utility (92, 93). Highly intrusive technologies, such as visual surveillance, are likely to provoke resistance among residents. Therefore, it is important to explore privacy-preserving and unobtrusive alternatives (e.g., radar sensing, smart floors, and acoustic analysis) (94). Furthermore, ensuring the security of the vast amounts of personal health and behavioral data collected, clarifying ownership and usage boundaries, and preventing misuse and discrimination pose significant challenges for the future development and application of AI technologies.

## 5 Conclusion

From an architectural perspective, this scoping review systematically integrates two dimensions, the LTCF building lifecycle stages and AI application pathways, into a two-dimensional analytical matrix, thereby providing a clear representation of the distribution of AI applications across different lifecycle stages. This framework moves beyond existing classification approaches that primarily organize studies by technology type, care service, or single design stage. Instead, it provides a cross-stage analytical perspective for identifying the coverage of AI applications and helps connect data flows and decision feedback across planning, design, operation, and assessment. The findings indicate that AI is promoting the intelligent transformation of LTCF built environments through a series of systematic methods. These applications primarily enhance technical and operational performance in terms of safety, comfort, energy efficiency, and adaptability, and may offer a potential pathway for translating human-centered design principles into spatial and operational practices in response to the challenges of population aging. Furthermore, as AI is increasingly applied to care resource allocation and service coordination, its relevance may extend beyond individual facility performance to the broader long-term care system, supporting service accessibility, equitable resource allocation, and continuity of care. It is worth noting that, although the analytical framework developed in this review is based on LTCFs, its lifecycle-oriented perspective may also provide a useful reference for systematically analyzing AI applications in other healthcare and care facilities.

Nevertheless, this study has several limitations. First, the literature search was mainly conducted in English and Chinese databases, which may have led to the omission of relevant studies published in other languages. Second, the included sources varied considerably in study design, data type, building context, and application scenario. Given this heterogeneity and the mapping purpose of a scoping review, the evidence was synthesized qualitatively. The review did not undertake a formal assessment of methodological quality or risk of bias, nor did it conduct a quantitative meta-analysis. Accordingly, this review characterizes the scope, distribution, and application domains of the available evidence rather than establishing comparative effectiveness or the overall strength of evidence. Third, most included studies primarily evaluated technical and operational indicators such as predictive accuracy, spatial efficiency, energy consumption, and activity- and fall-recognition performance, while relatively few have directly examined resident-level outcomes such as psychological well-being, dignity, and quality of care. Evidence regarding AI’s impact on older residents’ quality of life remains insufficient, and further research is needed to address this gap.

Based on these findings, future research can focus on the following areas. First, developing standardized data protocols and building high-quality, specialized databases covering the entire building lifecycle, thereby laying the foundation for model training and evaluation. Second, developing AI models capable of understanding and adapting to the complexity and diversity of LTCFs, taking into full account varying architectural conditions, physical and cognitive abilities, and dynamic care needs, to enhance the models’ adaptability and generalizability across different scenarios, particularly by integrating digital twin technologies to move from generic solutions toward personalized customization. Third, exploring low-cost, easy-to-deploy, and lightweight AI solutions to facilitate the application of AI technology in care facilities of varying levels across different regions (95). Fourth, advancing theories and standards for the coordinated integration of AI technologies and architectural space to create systematic, smart LTCF environments. Empowered by diverse AI technologies, future LTCFs will be better able to balance efficient professional care with a dignified residential experience, providing robust spatial infrastructure and technical support for addressing global aging challenges and enabling older adults to enjoy dignified and high-quality later lives.

## Data Availability

The original contributions presented in the study are included in the article/Supplementary material, further inquiries can be directed to the corresponding author.

## Glossary

2SFCA: Two-Step Floating Catchment Area
AAL: Ambient Assisted Living
ABM: Agent-Based Modeling
ABS: Agent-Based Simulation
ACO: Ant Colony Optimization
AI: Artificial Intelligence
AIP: Aging in Place
ANFIS: Adaptive Neuro-Fuzzy Inference System
ANN: Artificial Neural Network
BIM: Building Information Modeling
CCL: Connected Component Labeling
CGAN: Conditional Generative Adversarial Network
CNKI: China National Knowledge Infrastructure
CNN: Convolutional Neural Network
DGBO: Distribution-Guided Bayesian Optimization
DT: Decision Tree
FER-2013: Facial Expression Recognition 2013 Dataset
GA: Genetic Algorithm
GBDT: Gradient Boosting Decision Tree
GIS: Geographic Information System
HVAC: Heating, Ventilation, and Air Conditioning
ID3: Iterative Dichotomiser 3
IEQ: Indoor Environmental Quality
IoT: Internet of Things
LabVIEW: Laboratory Virtual Instrument Engineering Workbench
LLM: Large Language Model
LTC: Long-Term Care
LTCF/LTCFs: Long-Term Care Facility/Long-Term Care Facilities
ML: Machine Learning
MLP: Multilayer Perceptron
NSGA-II: Non-Dominated Sorting Genetic Algorithm II
POE: Post-Occupancy Evaluation
POI: Point of Interest
PRISMA-ScR: Preferred Reporting Items for Systematic Reviews and Meta-Analyses Extension for Scoping Reviews
RF: Random Forest
RL: Reinforcement Learning
RFID: Radio-Frequency Identification
SD: Stable Diffusion
SHAP: Shapley Additive Explanations
SVM: Support Vector Machine
XAI: Explainable Artificial Intelligence
XGBoost: Extreme Gradient Boosting
ADL/ADLs: Activity/Activities of Daily Living
EDGM: Emotional Dialogue Generation Model
FMCW: Frequency-Modulated Continuous-Wave
KNN: K-Nearest Neighbors
LoRA: Low-Rank Adaptation
