# Supplementary material for: Artificial intelligence applications in long-term care facilities: a scoping review across planning, design, operation, and assessment
Source: Front Public Health. 2026 Oct 19;14:1974526. doi: 10.3389/fpubh.2026.1974526 (PMC13724337; doi:10.3389/fpubh.2026.1974526)
Supplement: Supplementary file 1 [file Table_1.docx]

**Supplementary Materials**

**Table 1.** Database-specific search strings

NOTE: Targeted searches constituted the primary search strategy, while broader AI- and LTCF-related searches were additionally conducted as a supplementary approach to identify potentially relevant studies that might not be captured by lifecycle-specific terminology.

| **Database** | **Search Type** | **Search Strategy** |
| --- | --- | --- |
| **Web of Science** | **Broad search** | TS= ("Artificial Intelligence" OR "AI" OR "machine learning" OR "deep learning" OR "computer vision" OR "smart" OR "ambient assisted living" OR "AAL") AND TS= ("Long-term Care Facilities" OR "nursing home" OR "senior care facilities" OR "elderly care facilities" OR "community-embedded elderly care facilities") |
|  | **Targeted search**  **(Planning)** | TS= ("Artificial Intelligence" OR "AI" OR "machine learning" OR "deep learning" OR "computer vision" OR "smart" OR "ambient assisted living" OR "AAL") AND TS= ("Long-term Care Facilities" OR "nursing home" OR "senior care facilities" OR "elderly care facilities" OR "community-embedded elderly care facilities") AND TS= ("Planning" OR "location planning" OR "spatial distribution" OR "accessibility" OR "spatial equity") |
|  | **Targeted search**  **(Design)** | TS= ("Artificial Intelligence" OR "AI" OR "machine learning" OR "deep learning" OR "computer vision" OR "smart" OR "ambient assisted living" OR "AAL") AND TS= ("Long-term Care Facilities" OR "nursing home" OR "senior care facilities" OR "elderly care facilities" OR "community-embedded elderly care facilities") AND TS= ("Design" OR "spatial configuration" OR "spatial layout" OR "room layout" OR "interior design" OR "generative design") |
|  | **Targeted search**  **(Operation)** | TS= ("Artificial Intelligence" OR "AI" OR "machine learning" OR "deep learning" OR "computer vision" OR "smart" OR "ambient assisted living" OR "AAL") AND TS= ("Long-term Care Facilities" OR "nursing home" OR "senior care facilities" OR "elderly care facilities" OR "community-embedded elderly care facilities") AND TS= ("Operation" OR "management" OR "fall detection" OR "activity recognition" OR "behavior recognition" OR "safety monitoring" OR "environmental control" OR "indoor environment") |
|  | **Targeted search (Assessment and Retrofit)** | TS= ("Artificial Intelligence" OR "AI" OR "machine learning" OR "deep learning" OR "computer vision" OR "smart" OR "ambient assisted living" OR "AAL") AND TS= ("Long-term Care Facilities" OR "nursing home" OR "senior care facilities" OR "elderly care facilities" OR "community-embedded elderly care facilities") AND TS= ("Assessment and Retrofit" OR "evaluation" OR "safety assessment" OR "performance assessment" OR "post-occupancy evaluation" OR "POE" OR "age-friendly retrofitting" OR "renovation" OR "renewal" OR "refurbishment") |
| **Scopus** | **Broad search** | TITLE-ABS-KEY ("Artificial Intelligence" OR "AI" OR "machine learning" OR "deep learning" OR "computer vision" OR "smart" OR "ambient assisted living" OR "AAL") AND TITLE-ABS-KEY ("Long-term Care Facilities" OR "nursing home" OR "senior care facilities" OR "elderly care facilities" OR "community-embedded elderly care facilities") |
|  | **Targeted search (Planning)** | TITLE-ABS-KEY ("Artificial Intelligence" OR "AI" OR "machine learning" OR "deep learning" OR "computer vision" OR "smart" OR "ambient assisted living" OR "AAL") AND TITLE-ABS-KEY ("Long-term Care Facilities" OR "nursing home" OR "senior care facilities" OR "elderly care facilities" OR "community-embedded elderly care facilities") AND TITLE-ABS-KEY ("Planning" OR "location planning" OR "spatial distribution" OR "accessibility" OR "spatial equity") |
|  | **Targeted search**  **(Design)** | TITLE-ABS-KEY ("Artificial Intelligence" OR "AI" OR "machine learning" OR "deep learning" OR "computer vision" OR "smart" OR "ambient assisted living" OR "AAL") AND TITLE-ABS-KEY ("Long-term Care Facilities" OR "nursing home" OR "senior care facilities" OR "elderly care facilities" OR "community-embedded elderly care facilities") AND TITLE-ABS-KEY ("Design" OR "spatial configuration" OR "spatial layout" OR "room layout" OR "interior design" OR "generative design") |
|  | **Targeted search (Operation)** | TITLE-ABS-KEY ("Artificial Intelligence" OR "AI" OR "machine learning" OR "deep learning" OR "computer vision" OR "smart" OR "ambient assisted living" OR "AAL") AND TITLE-ABS-KEY ("Long-term Care Facilities" OR "nursing home" OR "senior care facilities" OR "elderly care facilities" OR "community-embedded elderly care facilities") AND TITLE-ABS-KEY ("Operation" OR "management" OR "fall detection" OR "activity recognition" OR "behavior recognition" OR "safety monitoring" OR "environmental control" OR "indoor environment") |
|  | **Targeted search (Assessment and Retrofit)** | TITLE-ABS-KEY ("Artificial Intelligence" OR "AI" OR "machine learning" OR "deep learning" OR "computer vision" OR "smart" OR "ambient assisted living" OR "AAL") AND TITLE-ABS-KEY ("Long-term Care Facilities" OR "nursing home" OR "senior care facilities" OR "elderly care facilities" OR "community-embedded elderly care facilities") AND TITLE-ABS-KEY ("Assessment and Retrofit" OR "evaluation" OR "safety assessment" OR "performance assessment" OR "post-occupancy evaluation" OR "POE" OR "age-friendly retrofitting" OR "renovation" OR "renewal" OR "refurbishment") |
| **Google Scholar** | **Broad search** | ("Artificial Intelligence" OR "AI" OR "machine learning" OR "deep learning" OR "computer vision" OR "smart" OR "ambient assisted living" OR "AAL") AND ("Long-term Care Facilities" OR "nursing home" OR "senior care facilities" OR "elderly care facilities" OR "community-embedded elderly care facilities") |
|  | **Targeted search (Planning)** | ("Artificial Intelligence" OR "AI" OR "machine learning" OR "deep learning" OR "computer vision" OR "smart" OR "ambient assisted living" OR "AAL") AND ("Long-term Care Facilities" OR "nursing home" OR "senior care facilities" OR "elderly care facilities" OR "community-embedded elderly care facilities") AND ("Planning" OR "location planning" OR "spatial distribution" OR "accessibility" OR "spatial equity") |
|  | **Targeted search**  **(Design)** | ("Artificial Intelligence" OR "AI" OR "machine learning" OR "deep learning" OR "computer vision" OR "smart" OR "ambient assisted living" OR "AAL") AND ("Long-term Care Facilities" OR "nursing home" OR "senior care facilities" OR "elderly care facilities" OR "community-embedded elderly care facilities") AND ("Design" OR "spatial configuration" OR "spatial layout" OR "room layout" OR "interior design" OR "generative design") |
|  | **Targeted search (Operation)** | ("Artificial Intelligence" OR "AI" OR "machine learning" OR "deep learning" OR "computer vision" OR "smart" OR "ambient assisted living" OR "AAL") AND ("Long-term Care Facilities" OR "nursing home" OR "senior care facilities" OR "elderly care facilities" OR "community-embedded elderly care facilities") AND ("Operation" OR "management" OR "fall detection" OR "activity recognition" OR "behavior recognition" OR "safety monitoring" OR "environmental control" OR "indoor environment") |
|  | **Targeted search (Assessment and Retrofit)** | ("Artificial Intelligence" OR "AI" OR "machine learning" OR "deep learning" OR "computer vision" OR "smart" OR "ambient assisted living" OR "AAL") AND ("Long-term Care Facilities" OR "nursing home" OR "senior care facilities" OR "elderly care facilities" OR "community-embedded elderly care facilities") AND ("Assessment and Retrofit" OR "evaluation" OR "safety assessment" OR "performance assessment" OR "post-occupancy evaluation" OR "POE" OR "age-friendly retrofitting" OR "renovation" OR "renewal" OR "refurbishment") |
| **SAGE Journals** | **Broad search** | ("Artificial Intelligence" OR "AI" OR "machine learning" OR "deep learning" OR "computer vision" OR "smart" OR "ambient assisted living" OR "AAL") AND ("Long-term Care Facilities" OR "nursing home" OR "senior care facilities" OR "elderly care facilities" OR "community-embedded elderly care facilities") |
|  | **Targeted search (Planning)** | ("Artificial Intelligence" OR "AI" OR "machine learning" OR "deep learning" OR "computer vision" OR "smart" OR "ambient assisted living" OR "AAL") AND ("Long-term Care Facilities" OR "nursing home" OR "senior care facilities" OR "elderly care facilities" OR "community-embedded elderly care facilities") AND ("location planning" OR "spatial distribution" OR "accessibility" OR "spatial equity") |
|  | **Targeted search**  **(Design)** | ("Artificial Intelligence" OR "AI" OR "machine learning" OR "deep learning" OR "computer vision" OR "smart" OR "ambient assisted living" OR "AAL") AND ("Long-term Care Facilities" OR "nursing home" OR "senior care facilities" OR "elderly care facilities" OR "community-embedded elderly care facilities") AND ("spatial design" OR "spatial configuration" OR "spatial layout" OR "room layout" OR "interior design" OR "generative design") |
|  | **Targeted search (Operation)** | ("Artificial Intelligence" OR "AI" OR "machine learning" OR "deep learning" OR "computer vision" OR "smart" OR "ambient assisted living" OR "AAL") AND ("Long-term Care Facilities" OR "nursing home" OR "senior care facilities" OR "elderly care facilities" OR "community-embedded elderly care facilities") AND ("fall detection" OR "activity recognition" OR "behavior recognition" OR "safety monitoring" OR "environmental control" OR "indoor environment") |
|  | **Targeted search (Assessment and Retrofit)** | ("Artificial Intelligence" OR "AI" OR "machine learning" OR "deep learning" OR "computer vision" OR "smart" OR "ambient assisted living" OR "AAL") AND ("Long-term Care Facilities" OR "nursing home" OR "senior care facilities" OR "elderly care facilities" OR "community-embedded elderly care facilities") AND ("safety assessment" OR "performance assessment" OR "post-occupancy evaluation" OR "POE" OR "age-friendly retrofitting" OR "renovation" OR "renewal" OR "refurbishment") |
| **CNKI** | **Broad search** | SU= (人工智能 OR AI OR 机器学习 OR 深度学习 OR 计算机视觉 OR 智能 OR 环境辅助生活) AND SU= (长期照护设施 OR 养老院 OR 养老机构 OR 养老设施OR 老年照护设施 OR 社区嵌入式养老设施) |
|  | **Targeted search (Planning)** | SU= (人工智能 OR AI OR 机器学习 OR 深度学习 OR 计算机视觉 OR 智能 OR 环境辅助生活) AND SU= (长期照护设施 OR 养老院 OR 养老机构 OR 养老设施OR 老年照护设施 OR 社区嵌入式养老设施) AND SU= (规划 OR 选址 OR 空间分布 OR 可达性 OR 空间公平) |
|  | **Targeted search**  **(Design)** | SU= (人工智能 OR AI OR 机器学习 OR 深度学习 OR 计算机视觉 OR 智能 OR 环境辅助生活) AND SU= (长期照护设施 OR 养老院 OR 养老机构 OR 养老设施 OR 老年照护设施 OR 社区嵌入式养老设施) AND SU= (设计 OR 空间配置 OR 空间布局 OR 房间布局 OR 室内设计 OR 生成式设计) |
|  | **Targeted search (Operation)** | SU= (人工智能 OR AI OR 机器学习 OR 深度学习 OR 计算机视觉 OR 智能 OR 环境辅助生活) AND SU= (长期照护设施 OR 养老院 OR 养老机构 OR 养老设施OR 老年照护设施 OR 社区嵌入式养老设施) AND SU= (运营 OR 管理 OR 跌倒检测 OR 活动识别 OR 行为识别 OR 安全监测 OR 环境控制 OR 室内环境) |
|  | **Targeted search (Assessment and Retrofit)** | SU= (人工智能 OR AI OR 机器学习 OR 深度学习 OR 计算机视觉 OR 智能 OR 环境辅助生活) AND SU= (长期照护设施 OR 养老院 OR 养老机构 OR 养老设施OR 老年照护设施 OR 社区嵌入式养老设施) AND SU= (评估与改造 OR 评价 OR 安全评估 OR 性能评估 OR 使用后评价 OR POE OR 适老化改造 OR 翻新 OR 更新 OR 整修) |
