# Supplementary material for: Artificial intelligence applications in long-term care facilities: a scoping review across planning, design, operation, and assessment
Source: Front Public Health. 2026 Oct 19;14:1974526. doi: 10.3389/fpubh.2026.1974526 (PMC13724337; doi:10.3389/fpubh.2026.1974526)
Supplement: Supplementary file 2 [file Table_2.docx]

**Supplementary Materials**

**Table 2.** Information of the 38 included studies

| **Ref.** | **Year** | **Country/**  **Regionᵃ** | **Core Method** | **Data Type** | **Outcome** |
| --- | --- | --- | --- | --- | --- |
| **Planning and Site Selection** | | | | | |
| (36) | 2024 | China | ID3 decision tree | POI data | Built a site-selection model with high prediction accuracy and proposed differentiated layout strategies for Guangzhou. |
| (37) | 2025 | China | ID3 decision tree | POI data | Suggested that suitable sites should prioritize proximity to government offices and medical institutions, while reducing subjective bias in site selection. |
| (38) | 2024 | China | ID3 decision tree | POI data;  Census data | Identified suitable spatial units for future elderly care facilities and clarified priority areas for construction. |
| (29) | 2025 | China | DT; RF; XGBoost;  SHAP explainability analysis | POI data;  Census data | Revealed the contribution of key spatial factors and identified priority areas for future facility provision. |
| (40) | 2024 | China | RF; GBDT | Multi-source spatial big data; Census data | Identified medical accessibility and older population distribution as key factors, and proposed differentiated site-selection recommendations. |
| (41) | 2023 | China | Improved NSGA-II | POI data;  Census data | Supported trade-off analysis among multiple objectives. |
| (47) | 2022 | China | Multi-agent simulation | Census data;  2019 Shanghai LTCF Dataset | Revealed spatial supply–demand mismatches and evaluated the spatial effects of policy interventions. |
| (46) | 2016 | Japan | ABM; ABS | GIS spatial data; Census data | Forecasted future demand for day-care centers and supported local-government planning strategies. |
| (42) | 2016 | China  (Hong Kong) | GA | GIS spatial data;  Population distribution data | Generated multiple Pareto-optimal plans and enabled planners to compare trade-offs among conflicting objectives. |
| (45) | 2023 | China | Spectral Clustering; Multinomial Logistic Regression | Questionnaire survey data; 2011–2020 Changsha LTCF Survey Data | Identified five demand-preference categories and proposed five allocation categories, with planning and subsidy recommendations. |
| **Architectural Design** | | | | | |
| (59) | 2024 | Canada | DGBO | Synthetic activity trajectories; Sensor-trigger data | Improved sensor-placement quality while reducing expensive simulator queries. |
| (57) | 2025 | Switzerland | ML | Isovist data;  Spatial topology features | Developed a framework that predicts dementia-friendly design performance using visual access indicators. |
| (49) | 2020 | UK | Self-organising GA; ABS; ACO algorithm | Spatial configuration data | Developed a generative optimization workflow for care-home layouts and supported performance-oriented floor-plan exploration. |
| (54) | 2024 | China | CGAN | LTC floor-plan image dataset | Generated zoning and floor-plan alternatives that support early design communication. |
| (50) | 2020 | Netherlands | A* pathfinding algorithm | Building boundary;  Daylight-hour simulation data; Spatial topology data | Developed a computational generation method balancing daylight potential and staff circulation efficiency. |
| (51) | 2024 | China | Path planning algorithm | Restroom floor plans; Simulated motion path data | Built an automated movement-path evaluation model and proposed layout improvements for accessible restrooms. |
| (48) | 2025 | China | GA | RFID data | Generated optimized functional-space allocation schemes better aligned with residents’ activity patterns. |
| (53) | 2025 | China | Long Short-Term Memory (LSTM); RL; CGAN | Multimodal sensor data; Behavior trajectories | Developed a closed-loop AI framework for aging-friendly transformation, improving spatial utilization, safety, and user satisfaction. |
| (52) | 2024 | China | SD + LoRA; GAN | Nursing-unit floor-plan images and boundary masks | Generated clear and readable functional block floor plans from nursing-unit boundaries. |

*(continued on next page)*

**Table 2 *(continued).***

| **Ref.** | **Year** | **Country/**  **Regionᵃ** | **Core Method** | **Data Type** | **Outcome** |
| --- | --- | --- | --- | --- | --- |
| **Operation and Management** | | | | | |
| (66) | 2025 | China | Attention-based LSTM; EDGM Fuzzy logic algorithm | Environmental perception data; Physiological feedback data | Proposed an interior design system that links environmental perception, emotional feedback, and adaptive spatial adjustment. |
| (65) | 2024 | Spain | ANN; MLP | Cooling-consumption monitoring data; Indoor/outdoor climate data | Built a high-accuracy model for predicting LTC cooling demand and informing energy-efficient thermal-comfort strategies. |
| (64) | 2018 | US | Multimodal depth and thermal sensing;  34-layer Residual Network (ResNet-34) CNN | Depth and thermal video data | Provided privacy-preserving activity monitoring and descriptive analytics for long-term health assessment. |
| (63) | 2011 | Canada | MLP | RGB video data with silhouette-derived velocity, area, and moment features | Detected simulated falls in real-world deployment and reduced false alarms using a prompting controller. |
| (67) | 2022 | China  (Taiwan) | CNN; MLP | Facial expression;  FER-2013 dataset; Environmental sensor data | Achieved 87% emotion-recognition accuracy and enabled real-time facial-emotion monitoring and potential environmental control in LTCFs. |
| (61) | 2022 | UK | SVM; KNN; CNN | FMCW radar micro-Doppler data of ADLs and falls | Developed a portable, privacy-preserving fall and ADL recognition system which can be deployed in LTCFs and is suitable for various indoor environments. |
| (62) | 2020 | US | ML-based classification; CCL | Floor pressure sensor-array signals | Developed a smart carpet system for fall detection, gait analysis, and activity monitoring without visual surveillance. |
| **Assessment and Retrofit** | | | | | |
| (75) | 2022 | Turkey | ANFIS | Expert risk-assessment scores; Spatial risk data from LTCFs | Built a hybrid expert system for classifying and quantifying spatial risks in LTCFs. |
| (73) | 2023 | Jordan | ANN; RF | IEQ measurements and satisfaction survey data | Validated high-accuracy ML models for IEQ satisfaction prediction and identified key environmental and architectural predictors. |
| (70) | 2020 | China | ML-based satisfaction model | IEQ measurements and satisfaction survey data | Found that air temperature had the strongest influence on overall IEQ satisfaction, followed by noise; provided evidence for design and operation decisions. |
| (79) | 2025 | China | NSGA-II | Sample building parametric model; Energy, thermal-comfort, and cost data | Developed a multi-objective retrofit framework and identified low-cost, comfort-oriented, and balanced retrofit strategies. |
| **Review Articles** | | | | | |
| (12) | 2021 | Italy | Review | N/A | Synthesized major AAL technologies, application contexts, and future challenges for healthy aging. |
| (13) | 2019 | China  (Hong Kong) | Review | N/A | Summarized technologies supporting cognition, mobility, social participation, and quality of life. |
| (16) | 2025 | Malaysia | Review | N/A | Mapped predictive analytics, XAI, multimodal sensing, privacy, ethics, and user-acceptance issues in ML-based AAL. |
| (14) | 2019 | US | Review | N/A | Showed potential for supporting independent living and care delivery, while emphasizing the need for developing customized technologies for different demographic groups in an aging society. |
| (10) | 2025 | China | Bibliometric analysis; VOSviewer; CiteSpace | N/A | Identified research hotspots such as machine learning and deep learning, and future trends toward personalized care and intelligent health management. |
| (11) | 2025 | China | Bibliometric analysis; VOSviewer; CiteSpace | N/A | Mapped research hotspots and emerging trends in age-friendly smart environment design. |
| (17) | 2022 | Netherlands | Review | N/A | Proposed design strategies for smart home modification, including customization, minimum life interference, and extensible technologies. |
| (15) | 2022 | Serbia | Review | N/A | Analyzed AI models, application domains, technological architectures, end users, and concerns such as privacy, transparency, usability, and acceptance. |

*ᵃ The geographical classification was based on the study setting or data source. In cases of multi-country collaborations or theoretical papers without a designated research site, the first author's institutional affiliation was used as the standard.*
